# Correlation of serum RBP4 level with oxidative stress and unstable carotid plaque in patients with cerebral infarction

**DOI:** 10.1515/tnsci-2022-0252

**Published:** 2022-10-10

**Authors:** Qingqing Wang, Sha Tian, Dahai Xiao, Ruotong Zhao, Xiaoxuan Zhang, Zhijie Dou, Chengbo Li, Zheng Ma

**Affiliations:** Department of Neurology, Chengde Medical University Affiliated Hospital, Hebei, China

**Keywords:** carotid atherosclerosis, cerebral infarction, oxidative stress, retinol-binding protein 4

## Abstract

**Objectives:**

This study aimed to investigate the changes in serum levels of retinol-binding protein 4 (RBP4) with cerebral infarction, relationship of RBP4 with oxidative stress and carotid atherosclerosis, and its possible role in cerebral infarction.

**Materials and methods:**

According to the results of cervical vascular ultrasound, the experimental group was divided into three groups: intima thickening group (*n* = 31), stable plaque group (*n* = 51), and unstable plaque group (*n* = 54). Forty healthy subjects were selected as the control group. Their serum levels of RBP4, 8-iso-prostaglandin-F2alpha (8-iso-PGF2α), and catalase (CAT) were measured. Carotid vascular ultrasound was used to measure the plaque area and intima-media thickness (IMT).

**Results:**

The serum RBP4 and 8-iso-PGF2α levels, IMT and plaque area in the control, intimal thickening, stable plaque, and unstable plaque groups increased, while the serum level of CAT decreased (*P* < 0.001). The serum levels of RBP4 positively correlated with 8-iso-PGF2α, IMT, and plaque area and negatively correlated with CAT level. The area under the receiver operating characteristic curve was 0.778 in predicting unstable plaques.

**Conclusions:**

The serum levels of RBP4 were significantly elevated in elderly patients with cerebral infarction and correlated with oxidative stress injury and the degree of atherosclerosis. Serum RBP4 has diagnostic value for unstable plaques in carotid arteries.

## 1 Introduction

The incidence and morbidity of cerebral infarction continue to increase with the aging of society and the improvement in people’s living standards, posing a serious threat to people’s health. Some studies have shown that 80% of patients with cerebral infarction are aged ≥60 years [1]. Atherosclerosis is the main pathogenesis of cerebral infarction. Oxidative stress injury plays an important role in the development of atherosclerosis [2–4].

Yang et al. [5]. found that retinol-binding protein 4 (RBP4) was a new adipocytokine belonging to the lipocalin family. It is essentially a lipophilic protein mainly synthesized by hepatocytes and adipose tissue. Besides being associated with obesity, hypertension, insulin resistance, and diabetes, RBP4 is also a risk factor for cardiovascular diseases, such as metabolic syndrome, atherosclerosis, and coronary heart disease [6–11]. Nicotinamide adenine dinucleotide phosphate oxidase and nuclear factor kappa-B activation lead to oxidative stress injury, inducing an inflammatory response in endothelial cells [12]. In recent years, elevated levels of RBP4 have been associated with carotid intima-media thickness (IMT) and atheromatous plaque circulation in patients with rheumatoid arthritis. It has been suggested that RBP4 plays a role in the development of atherosclerosis. However, the mechanism underlying its role in the development of atherosclerosis is unclear. Further, the relationship between serum levels of RBP4 and oxidative stress injury and the degree of atherosclerosis in elderly patients with cerebral infarction has not been reported in the literature. This study aimed to investigate the changes in serum RBP4 levels in elderly patients with cerebral infarction and their relationship with oxidative stress and carotid atherosclerotic plaques so as to provide a reference for clinical diagnosis and treatment.

## 2 Materials and methods

### 2.1 Participants

A total of 136 consecutive patients with cerebral infarction and 40 age- and sex-matched control participants were enrolled between August 2020 and June 2021 in the Affiliated Hospital of Chengde Medical College. The patients with cerebral infarction included 89 men and 47 women, with a mean age of 68.7 ± 5.3 years (range, 60–80 years). All 40 control participants (24 men and 16 women) had no history of cerebral events. They had a mean age of 66.4 ± 4.4 years (range, 60–80 years). Based on the results of the carotid ultrasound examination, the carotid artery was divided into intima thickening group (*n* = 31), plaque stability group (*n* = 51), and plaque instability group (*n* = 54).

The inclusion criteria were as follows: patients meeting the diagnostic criteria of the Chinese Guidelines for the Diagnosis and Treatment of Acute Ischemic Stroke 2018 and confirmed by cranial imaging, patients aged 60–80 years and with first cerebral infarction, time from onset to admission <72 h, and cervical vascular ultrasound received within 72 h of admission.

Patients with a history of recent (within the last 6 months) severe chronic heart failure (class NYHA II–IV), malignant diseases, major trauma or surgery, severe renal (creatinine >2 mg/dL) or liver insufficiency (ALT >2 times upper normal limit), acute or chronic infectious disease, hematological system disorders, or any kind of immune-mediated disease were excluded. Patients on statins, homocysteine-lowering (Hcy) drugs, and antioxidant vitamin supplements (e.g., vitamins E and C) for three consecutive months prior to admission were also excluded. Patients with diabetes and/or recent (within the last 6 months) use of glucose-lowering medications were also excluded.


**Ethical approval:** The research related to human use has been complied with all the relevant national regulations, institutional policies and in accordance the tenets of the Helsinki Declaration, and has been approved by the Medical Ethics Committee of the Affiliated Hospital of Chengde Medical College (Approval number: CYFYLL2021165). Signed informed consent forms were obtained from the patients or guardians.
**Informed consent:** Informed consent has been obtained from all individuals included in this study.

### 2.2 Measurements

A questionnaire was used to obtain general clinical information about the patients, such as age, sex, body mass index (BMI), smoking, alcohol consumption, and past history (e.g., hypertension, diabetes, and coronary artery disease).

Determination of biochemical indicators: fasting peripheral blood (10 mL) was drawn from all included patients after fasting for solids and liquids for 10 h overnight. The upper serum was used to determine biochemical indicators, including glycosylated hemoglobin A1c (HbA1c) level measured using a glycosylated hemoglobin analyzer, and levels of total cholesterol (TC), triglyceride (TG), low-density lipoprotein cholesterol (LDL-C), and high-density lipoprotein cholesterol (HDL-C) measured using an automatic biochemical analyzer.

Measurement of serum levels of RBP4, 8-iso-prostaglandin-F2alpha (8-iso-PGF2α), and catalase (CAT): the enrolled patients did not eat or drink for 10 h overnight, 3 mL of the peripheral blood was drawn, and the serum was collected. The serum was centrifuged at 3,000 rpm for 10 min, and the supernatant was removed and placed in a –80°C refrigerator for freezing and storage. The serum RBP4, 8-iso-PGF2α, and CAT levels were detected using the enzyme-linked immunosorbent assay. The reagents were provided by the Wuhan Huamei Company.

Within 72 h of admission, all participants were examined by an experienced attending carotid ultrasonographer in a lying position with the patient’s head tilted to one side, using a SonSite Edge color Doppler ultrasonograph (probe frequency 10 MHz) to investigate the common carotid artery, the bifurcation of the carotid artery, the beginning of the internal carotid artery, and the external carotid artery bilaterally.

Evaluation of the carotid artery: the IMT of the carotid arteries was measured three times, and the average value was taken as the final measurement result. The carotid plaques were classified according to the test results: (1) normal carotid intima-media: IMT ≤1.0 mm; (2) carotid intima-media thickening: IMT >1.0 mm and IMT <1.2 mm; (3) carotid stable plaque: carotid visible plaque, IMT ≥1.0 mm and IMT < 2.0 mm, showing hyperechoic plaque or isoechoic plaque, but not consistent with any sign of unstable plaque; and (4) carotid unstable plaque: IMT ≥2.0 mm, or at least one sign of carotid unstable plaque, including hypoechoic or heterogeneous echogenicity.

### 2.3 Statistical analysis

The computer statistical software package SPSS24.0 was used for data analysis. The measurement data conforming to normal distribution were expressed as mean values ± standard deviation, and those not conforming to normal distribution were expressed as M (Q1, Q2). One-way analysis of variance was used for analyzing variance, and the rank-sum test was used for variance disparity. The count data were expressed as frequencies or percentages, and the *χ* ^2^ test was used to compare enumeration data. Pearson correlation analysis was used for the correlation test. Receiver operating characteristic (ROC) curve was used to assess the best cutoff point for RBP4 to predict the presence of unstable carotid plaques in elderly patients with cerebral infarction. A *P* value <0.05 indicated a statistically significant difference.

## 3 Results

### 3.1 General clinical data

General clinical data such as sex, age, BMI, hypertension, diabetes mellitus, smoking, and alcohol consumption were compared among the groups showing no statistically significant differences (*P* > 0.05) (Table 1).

**Table 1:** Comparison of general clinical data and laboratory indices between groups

	Control	Intima thickening group	Plaque stability group	Plaque instability group	*P-*value
Participants, *n*	40	31	51	54	
Sex, M/F	24/16	9/22	31/20	36/18	0.723
Age (year)	66.38 ± 4.43	68.45 ± 5.69	68.10 ± 5.14	69.30 ± 5.13	0.056
BMI (kg/m^2^)	23.7 ± 4.3	25.3 ± 3.0	24.6 ± 3.7	23.4 ± 2.6	0.062
Hypertension, *n* (%)	22 (55)	21 (67.7)	38 (74.5)	41 (75.9)	0.13
Diabetes mellitus, *n* (%)	7 (17.5)	11 (35.5)	17 (33.3)	15 (27.8)	0.293
Smoking history, *n* (%)	19 (47.5)	18 (58.1)	29 (56.9)	28 (51.9)	0.77
Drinking history, *n* (%)	18 (45)	16 (51.6)	27 (52.9)	33 (61.1)	0.481
WBC (10^9^/L)	8.2 ± 2.0	7.7 ± 2.8	7.2 ± 2.8	7.5 ± 2.4	0.288
Hcy (µmol/L)	15.3 (12.6–22.0)	15.0 (11.6–22.2)	15.0 (11.6–20.0)	16.2 (513.0–21.5)	0.53
HbA1c (％)	5.6 (5.3–6.0)	5.9 (5.5–7.3)	5.7 (5.4–7.1)	5.7 (5.3–6.7)	0.692
HDL-C (mmol/L)	1.1 ± 0.3	1.1 ± 0.4	1.1 ± 0.3	1.1 ± 0.2	0.982
LDL-C (mmol/L)	2.3 ± 0.8	2.2 ± 0.9	2.3 ± 10.0	2.5 ± 10.0	0.436
TC (mmol/L)	4.2 ± 0.9	4.4 ± 0.9	4.2 ± 1.1	4.4 ± 1.1	0.59
TG (mmol/L)	1.7 ± 0.7	2.1 ± 1.0	1.8 ± 0.8	1.7 ± 0.8	0.24
RBP4 (mg/L)	13.8 ± 6.5	19.3 ± 9.1^a^	24.9 ± 9.1^ab^	29.7 ± 8.7^abc^	<0.001
8-iso-PGF2α (pg/mL)	173.9 ± 60.8	254.4 ± 81.8^a^	271.0 ± 75.3^a^	364.6 ± 89.8^abc^	<0.001
CAT (ng/mL)	71.3 ± 33.2	49.8 ± 32.3^a^	47.2 ± 27.4^a^	41.5 ± 25.8^a^	<0.001
IMT (mm)	0.5 ± 0.1	1.1 ± 0.1^a^	1.6 ± 0.2^ab^	3.0 ± 0.5^abc^	<0.001
Plaque area (mm^2^)	5.9 ± 2.5	6.0 ± 2.2	30.4 ± 15.7^ab^	33.3 ± 18.0^ab^	<0.001

Note: ^a^Compared with the control group, *P* < 0.01. ^b^Compared with the intima thickening group, *P* < 0.01. ^c^Compared with the plaque stability group, *P* < 0.01.

### 3.2 Comparison of indexes in each group

**Figure 1:**
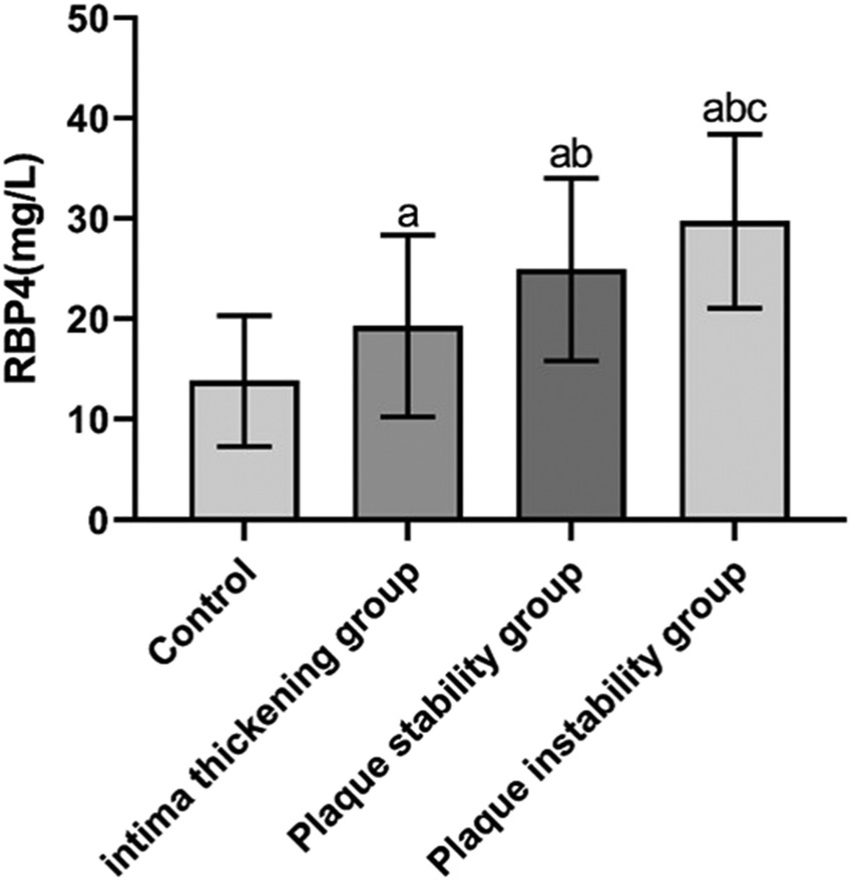
RBP4 concentrations in the study population.

**Figure 2:**
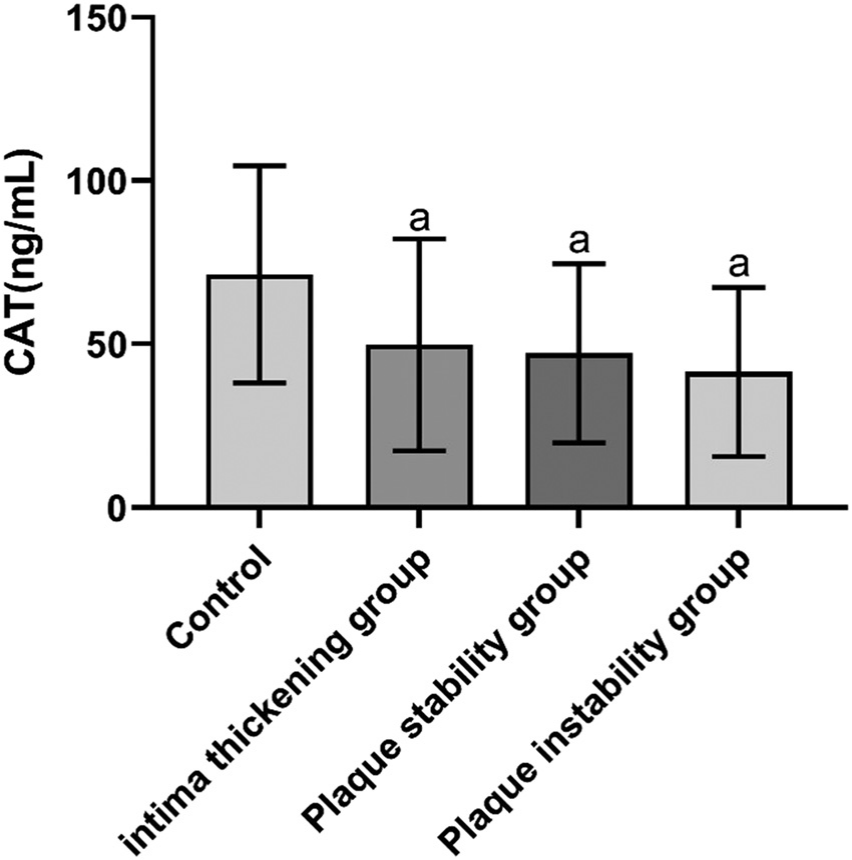
CAT concentrations in the study population.

**Figure 3:**
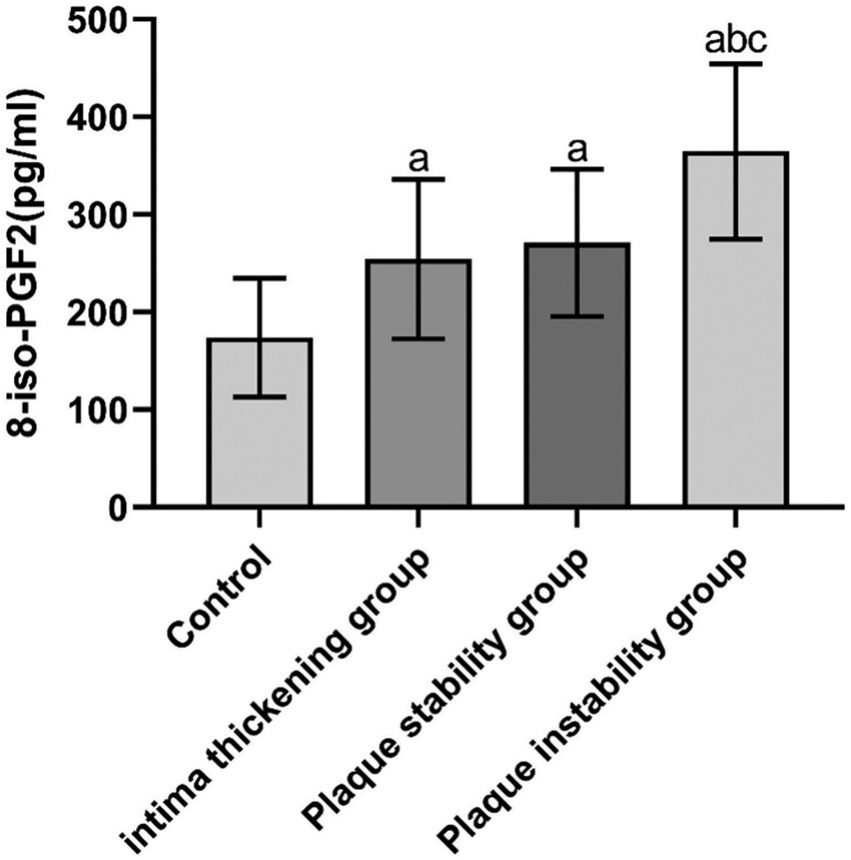
8-iso-PGF2α concentrations in the study population.

The differences were not statistically significant when the levels of TG, TC, HDL-C, LDL-C, HbA1c, Hcy, and white blood cell (WBC) count in each group (*P* > 0.05) were compared. The differences were statistically significant when the levels of RBP4, 8-iso-PGF2α, CAT, IMT, and carotid plaque area were compared in the control, intimal thickening, stable plaque, and unstable plaque groups (*P* < 0.05 and *P* < 0.01). The levels of RBP4, 8-iso-PGF2α, CAT, IMT and carotid plaque area were significantly higher in the intimal thickening, stable plaque, and unstable plaque group, compared with the control group, and the difference was statistically significant (*P* < 0.01). The levels of RBP4, IMT, and carotid plaque area in the stable and unstable plaque groups and the 8-iso-PGF2α level in the unstable plaque group were higher than those in the intimal thickening group, and the differences were statistically significant (*P* < 0.01). The levels of RBP4, 8-iso-PGF2α, and IMT were higher in the unstable plaque group than that in the stable plaque group, and the differences were statistically significant (*P* < 0.01) (Table 1 and Figures 1–3).

### 3.3 Comparison of serum levels of RBP4 with oxidative stress

Serum levels of RBP4 and 8-iso-PGF2α were significantly higher, whereas serum level of CAT was significantly lower in patients with cerebral infarction compared with controls (*P* < 0.01) (Table 1 and Figures 1–3).

### 3.4 Comparison of carotid atherosclerotic indexes

The carotid plaque area and IMT were significantly higher in elderly patients with cerebral infarction than in the controls (*P* < 0.01) (Table 1).

### 3.5 Correlation of the serum level of RBP4 with oxidative stress and carotid atherosclerosis

**Figure 4:**
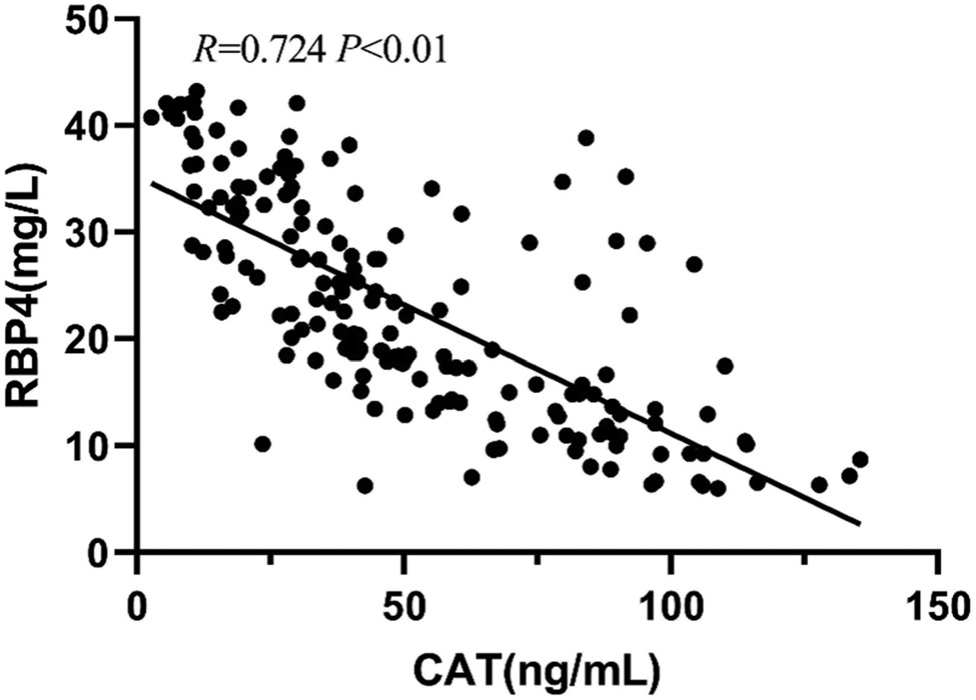
Correlation between the levels of RBP4 and CAT.

**Figure 5:**
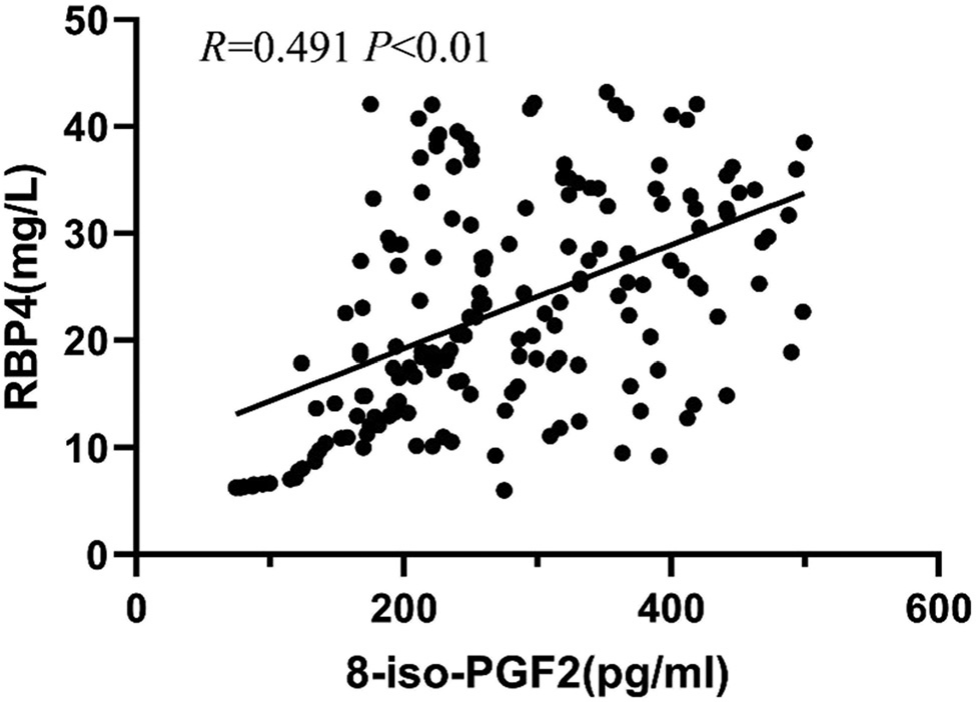
Correlation between the levels of RBP4 and 8-iso-PGF2α.

**Figure 6:**
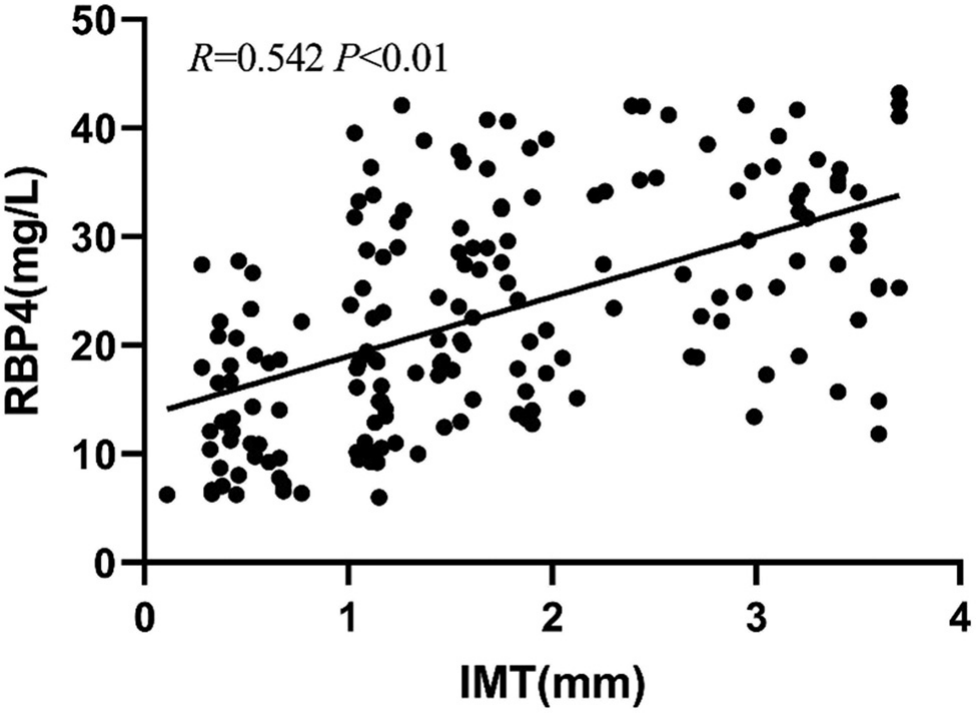
Correlation between RBP4 level and IMT.

**Figure 7:**
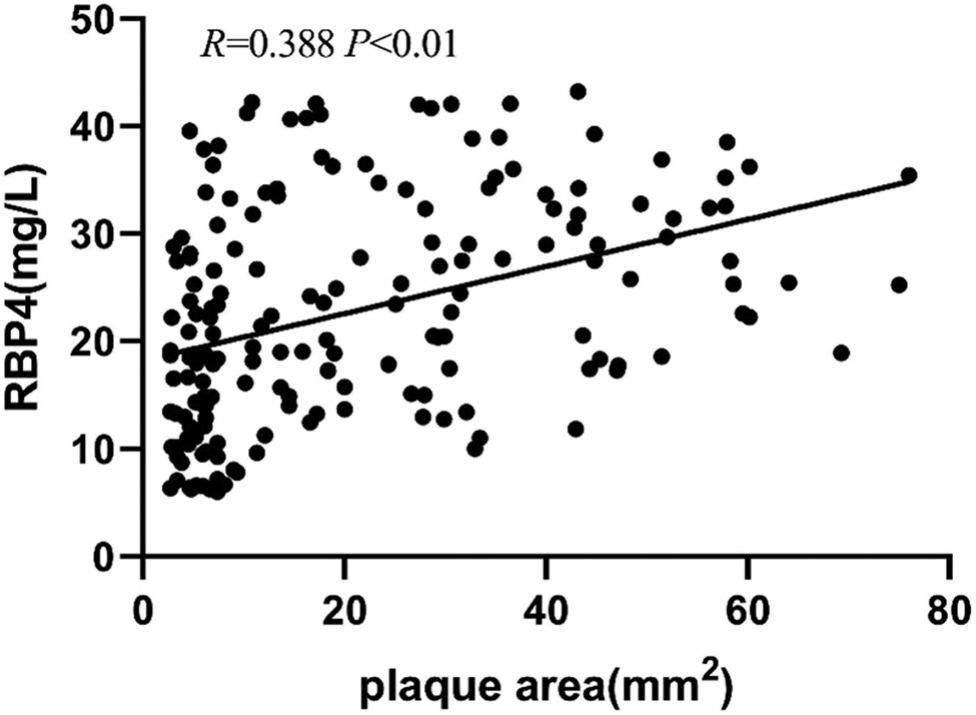
Correlation between RBP4 level and plaque area.

Pearson correlation analysis showed that the serum level of RBP4 positively correlated with the 8-iso-PGF2α level, IMT, and carotid plaque area (*P* < 0.01) but negatively correlated with the serum level of CAT (*P* < 0.01) (Table 2 and Figures 4–7).

**Table 2:** Correlation analysis of baseline data and RBP4 levels

Parameter	*r*	*P*
CAT (ng/mL)	–0.724	0.000
8-iso-PGF2α (pg/mL)	0.491	0.000
IMT (mm)	0.542	0.000
Plaque area (mm^2^)	0.388	0.000

### 3.6 ROC curve analysis

**Figure 8:**
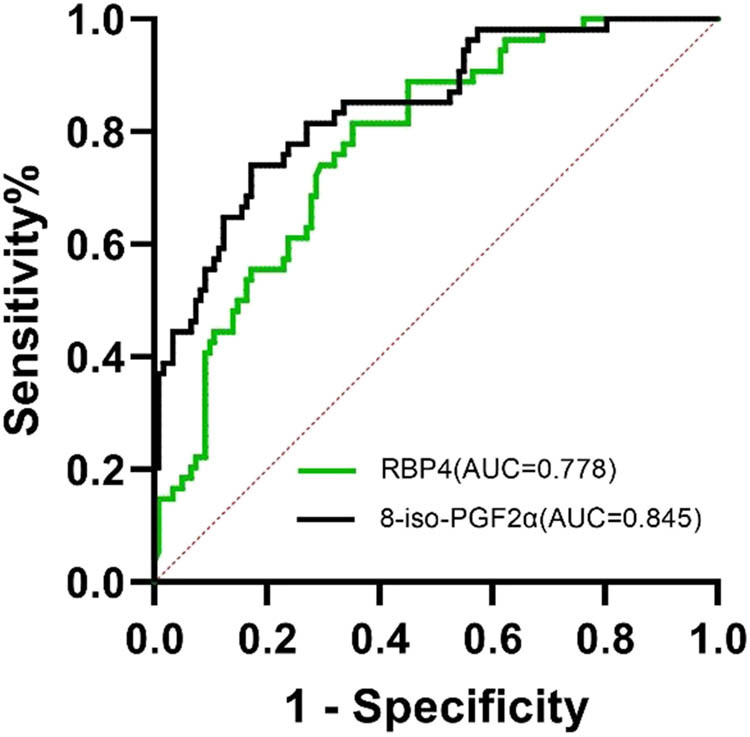
ROC curve analysis of RBP4 (green line) and 8-iso-PGF2α (black line) as markers for diagnosing unstable carotid plaques.

The optimal cutoff point for the serum level of RBP4 to predict the occurrence of unstable plaques in carotid arteries was 22.215 mg/L, and the area under the ROC curve was 0.778 (95% CI: 0.708–0.848, *P* = 0.000), with a sensitivity of 81.5% and specificity of 64.8%. The optimal cutoff point for the serum level of 8-iso-PGF2α to predict the occurrence of unstable plaques in carotid arteries was 316.74 mg/L, and the area under the ROC curve was 0.845 (95% CI: 0.782–0.908, *P* = 0.000), with a sensitivity of 74.1% and specificity of 82.8% (Table 3 and Figure 8).

**Table 3:** ROC curve of RBP4 and 8-iso-PGF2α in the diagnosis of unstable plaques in the carotid artery

Projects	Cutoff	AUC	Sensitivity (%)	Specificity (%)	Youden index	*P*	95% CI
RBP4 (mg/L)	22.215	0.778	0.815	0.648	0.463	0.000	0.708–0.848
8-iso-PGF2α (pg/mL)	316.74	0.845	0.741	0.828	0.569	0.000	0.782–0.908

## 4 Discussion

Nowadays, stroke is a major problem affecting the entire world. It is characterized by high morbidity, mortality, disability, and recurrence. A study published in *The Lancet* in 2019 showed that stroke was the number one disease causing death and disability in China [13]. Carotid atherosclerosis is the most important pathophysiological basis of cerebral infarction. RBP4 is a novel adipokine; however, the exact mechanism of its involvement in cerebral infarction is not fully understood. Liu et al. [14] found that the expression level of RBP4 increased in atherosclerotic plaque tissues in both patients with atherosclerosis and in mouse models. They also found that the expression of the RBP4 gene promoted macrophage-derived foam cell formation and accelerated the process of atherosclerosis. Most scholars used IMT of carotid arteries as a measure of atherosclerotic burden due to the safety, economy, and feasibility of ultrasound devices [15]. In addition, one study pointed out that higher RBP4 levels led to oxidative damage [16]. One of the early and most important components of cerebral infarction is reactive oxygen species-induced tissue damage. The brain is highly susceptible to oxidative stress-induced damage due to its complex structure, relatively low antioxidant capacity, high oxidative metabolic activity, insufficient neuronal cell repair activity, and high polyunsaturated fatty acid content [17]. Dysfunction of the antioxidant system is the main mechanism causing the generation of oxidative stress. CAT is an important antioxidant enzyme that scavenges free radicals in the body, and the changes in the CAT level in the body can reflect the strength of the body’s resistance to oxidative stress. 8-iso-PGF2α is an end product of lipid peroxidation of unsaturated fatty acids. Its production is related to peroxidative damage and reflects the level of oxidative stress in the body. This study found that the serum level of RBP4 not only positively correlated with carotid artery IMT and atherosclerotic plaque area but also significantly positively correlated with the serum level of 8-iso-PGF2α and negatively correlated with CAT level in elderly patients with cerebral infarction. It suggested that the elevated level of RBP4 in elderly patients with cerebral infarction promoted oxidative stress injury, which might be one of the important mechanisms of occurrence and development of cerebral infarction.

In the present study, the serum levels of RBP4 were found to be higher in the intimal thickening, stable plaque, and unstable plaque groups than in the control group. The results of Sasaki et al. [18] showed that the plasma levels of RBP4 were higher in patients with acute ischemic stroke than in the controls, and suggested that elevated plasma levels of RBP4 could be used as a new biomarker for the development of acute ischemic stroke, which was consistent with the results of the present study. Similarly, serum levels of RBP4 were elevated in patients with coronary artery disease [19]. However, a study of middle-aged White women found that RBP4 levels were not significantly associated with an increased risk of stroke in women [20]. Populations, sample sizes, kits, and races might differ in these studies; this study was performed on an elderly population.

Unstable plaques are characterized by a thin fibrous cap, a large, soft lipid core, low collagen content, irregular morphology, more neovascular growth, and susceptibility to rupture [21]. Plaque rupture exposes lipids, which activate platelets and initiate coagulation reactions to form thrombi, leading to cerebral infarction. Previous studies showed that the more unstable the carotid plaques, the greater the risk of acute cerebral infarction [22]. In this study, the serum levels of RBP4 were found to be increased sequentially in the control, intimal thickening, stable plaque, and unstable plaque groups. Further analysis using the ROC curve revealed that the optimal cutoff point of the serum RBP4 level in predicting the occurrence of the carotid unstable plaque was 22.215 mg/L, the area under the ROC curve was 0.778, and the optimal cutoff point of the serum 8-iso-PGF2α level in predicting the occurrence of the carotid unstable plaque was 316.74 pg/mL. The area under the ROC curve was 0.845, both of which could well evaluate the occurrence of the carotid unstable plaque.

In conclusion, the serum levels of RBP4 were significantly elevated in elderly patients with cerebral infarction and correlated with oxidative stress injury and the degree of atherosclerosis. Serum RBP4 has a certain predictive value for carotid artery unstable plaque, which can guide the clinical development of timely and effective intervention measures. More in-depth molecular biological studies should be performed to elucidate the exact mechanism of RBP4 involvement in the development of cerebral infarction in elderly patients. This study had some limitations: (1) the sample size was not large enough since it was not a multicenter study and (2) color Doppler ultrasound still has certain limitations in evaluating the nature of atherosclerotic plaques.
